# Coronary Artery Disease and Atrial Fibrillation: A Bidirectional Mendelian Randomization Study

**DOI:** 10.3390/jcdd9030069

**Published:** 2022-02-27

**Authors:** Tao Yan, Shijie Zhu, Changming Xie, Miao Zhu, Fan Weng, Chunsheng Wang, Changfa Guo

**Affiliations:** 1 Department of Cardiovascular Surgery, Zhongshan Hospital, Fudan University, Shanghai 200032, China; 19211210038@fudan.edu.cn (T.Y.); zhu.shijie@zs-hospital.sh.cn (S.Z.); 20211210111@fudan.edu.cn (M.Z.); 21211210056@m.fudan.edu.cn (F.W.); 2 Department of Cardiovascular, The Eighth Affiliated Hospital, Sun Yat-sen University, Shenzhen 518033, China; xiechm3@mail2.sysu.edu.cn

**Keywords:** coronary artery disease, atrial fibrillation, Mendelian randomization, genetics

## Abstract

Background: Several works of observational clinical research indicate that coronary artery disease (CAD) and atrial fibrillation (AF) aggravate each other. However, it is unknown whether these associations reveal independent causal processes. Objective: The present study aimed to evaluate causal associations between CAD and AF using two-sample Mendelian randomization (TSMR) analysis. Methods: Summary-level Genome-wide association study (GWAS) data for CAD were obtained from the CARDIoGRAMplusC4D consortium, including 60,801 patients and 123,504 controls. General data for AF were acquired from the largest meta-analysis, comprising of 60,620 patients with AF and 970,216 non-cases. After data harmonization, three different methods—inverse-variance weighted (IVW), MR-Egger, and weighted-median—were applied for TSMR analysis. Results: The calculated ORs (95% CIs) for AF using IVW, MR-Egger, and weighted-median analysis were 1.11 (1.05, 1.17; *p*-value < 0.001), 1.14 (1.00, 1.29; *p*-value = 0.049), and 1.13 (1.08, 1.19; *p*-value < 0.001), respectively; for CAD, the results were 1.01 (0.97, 1.04; *p*-value = 0.76), 0.95 (0.89, 1.02; *p*-value = 0.15), and 1.00 (0.95, 1.05; *p*-value = 0.97). Conclusion: This comprehensive TSMR analysis provides evidence that patients with CAD are associated with an increased risk of AF. However, no causal association was found between patients with AF and the risk of CAD. These findings benefit clinical decision-making. Early heart-rhythm monitoring should be performed in patients with CAD. The prevention and treatment of AF complications such as thrombosis may be essential to reduce the incidence of CAD in AF patients.

## 1. Introduction

Cardiovascular diseases (CVDs), including coronary artery disease (CAD), atrial fibrillation (AF), and other cardiac and vascular diseases, remain leading contributors to death worldwide [1]. CAD is a common disease in the clinic, associated with a high sudden-death rate and adverse complications [2]. AF is the most common tachyarrhythmia globally [3], reducing the life quality of patients, and increasing the economic burden for both individuals and public health [4]. Increasing evidence demonstrates that a strong association exists between CAD and AF [5]. Several previous studies have shown that the incidence of AF is higher in CAD patients than in healthy people [6,7,8]. On the other hand, AF was identified as an independent risk factor of incident myocardial infarction [9]. The incidence of CAD in patients with AF was 34%, which was significantly higher than that of healthy people [10]. Although these clinical studies revealed the association between CAD and AF, the existence of some common risk factors such as hypertension, diabetes mellitus, and obesity, may bias the results. Furthermore, it is difficult to infer causation based solely on observational analyses, which remain potentially unmeasured confounders and reverse causality. In addition, a large-scale randomized clinical trial also requires more economical costs.

Mendelian randomization analysis is a method mainly used in epidemiological etiology inference in recent years, which uses instrumental variables (IVs) to infer the causal relationship between exposure and outcome [11]. In fact, different genotypes determine different intermediate phenotypes, which represent certain exposure characteristics of individuals. Therefore, the association between genotype and outcome can represent the effect of exposure on the outcome. Since alleles follow the principle of random allocation, this effect is not affected by confounders and reverse causality, which is an alternative approach to inferring the extent of causality.

Herein, we conducted a bidirectional two-sample Mendelian randomization (TSMR) analysis, for the first time, to assess causal associations between CAD and AF. 

## 2. Methods

### 2.1. Population Selection

General Genome-wide association study (GWAS) data for CAD were acquired from the Coronary ARtery DIsease Genome-wide Replication and Meta-analysis, plus The Coronary Artery Disease Genetics (CARDIoGRAMplusC4D) consortium, including 60,801 patients and 123,504 controls [12]. The most recent and largest meta-analysis was used to identify the genetic variation underlying AF, comparing 60,620 patients with AF and 970,216 non-cases from six contributing studies, including the Nord-Trøndelag Health Study (HUNT); the Diabetes Epidemiology: Collaborative analysis of Diagnostic criteria in Europe study (deCODE); the Michigan Genomics Initiative (MGI); DiscovEHR; UK Biobank; and the Atrial Fibrillation Genetics (AFGen) Consortium [13]. Due to the present study being a re-analysis of published data, no ethics approval was required. 

### 2.2. IVs Construction

IVs must satisfy three core assumptions: (1) they are significantly associated with the exposure of interest; (2) they are not related to any confounders of the exposure-outcome association; and (3) they affect the outcome only through exposure [14]. To meet these conditions, we first explored SNPs associated with exposure at GWAS significance with a *p*-value < 5 × 10^−8^. Afterward, a linkage disequilibrium clumping algorithm with *r*^2^ threshold = 0.001 was applied to ensure the independence of IVs for exposure. Directional horizontal pleiotropy was tested utilizing Mendelian-randomization-Egger (MR-Egger) regression analysis [15]. If the intercept of MR-Egger regression analysis had *p* > 0.05, it indicated that no horizontal pleiotropic pathway exists. 

### 2.3. Data Harmonization

To make sure that the effect of an SNP on the exposure and the outcome corresponded to the same allele, it was critical to perform data harmonization. If the alleles did not correspond for the same SNP, this SNP would be discarded from the analysis. For palindromic SNP, although the alleles corresponded, the alleles on the forward strand and the reverse strand were the same, so we tried to infer the forward strand alleles using allele frequency information. If any palindromic SNPs had an allele frequency of less than the threshold of 0.42, we would discard the SNPs, because the allele frequency would no longer give us information about the strand.

### 2.4. Statistical Analysis

TSMR analysis was performed to determine the causal associations between AF and CAD utilizing the TwoSampleMR package in R language. All GWAS analyses were corrected using the Bonferroni method. Three methods, including inverse-variance-weighted (IVW) [16], MR-Egger [17], and weighted-median [18], were used for TSMR to ensure the robustness of the analysis. The leave-one-out sensitivity analysis was then utilized to determine the impact of a single SNP on TSMR analysis [19]. 

## 3. Results

### 3.1. Causal Effect of CAD on AF

A total of 41 IVs were identified. The details of 41 IVs are shown in Table S1. After the data harmonization, two SNPs, rs10139550 and rs7568458, were removed because they were palindromic with intermediate allele frequencies. The intercept term from MR-Egger regression analysis demonstrated that no horizontal pleiotropic pathway existed in the TSMR analysis (Table 1). The calculated ORs (95% CIs) for AF using IVW, MR-Egger, and weighted-median analysis were 1.11 (1.05, 1.17; *p*-value < 0.001), 1.14 (1.00, 1.29; *p*-value = 0.049), and 1.13 (1.08, 1.19; *p*-value < 0.001), respectively, as shown in Table 2; this demonstrated that CAD might genetically increase the risk of AF. The leave-one-out sensitivity analysis also confirmed the impact of CAD on AF. In addition, considering that myocardial infarction (MI) subtype accounts for up to 70% of CAD, we further analyzed the association of MI on AF. Similarly, the calculated ORs (95% CIs) for MI on AF using IVW, MR-Egger, and weighted-median analysis were 1.11 (1.06, 1.16; *p*-value < 0.001), 1.23 (1.11, 1.37; *p*-value < 0.001), and 1.13 (1.07, 1.19; *p*-value < 0.001), respectively.

### 3.2. Causal Effect of AF on CAD

A total of 111 IVs were identified, as shown in Table S3. The SNP rs4965430 was discarded due to a lack of information about the strand. The MR-Egger regression analysis did not show a horizontal pleiotropic pathway in the TSMR analysis (Table 1). The calculated ORs (95% CIs) for CAD using IVW, MR-Egger, and weighted-median analysis were 1.01 (0.97, 1.04; *p*-value = 0.76), 0.95 (0.89, 1.02; *p*-value = 0.15), and 1.00 (0.95, 1.05; *p*-value = 0.97), respectively, as shown in Table 3. These results revealed no evidence of a causal effect of AF on CAD, genetically. The analysis of AF on the MI subtype showed the same results. The calculated ORs (95% CIs) for MI using IVW, MR-Egger, and weighted-median analysis were 1.00 (0.96, 1.04; *p*-value = 0.98), 0.94 (0.87, 1.01; *p*-value = 0.11), and 0.98 (0.92, 1.03; *p*-value = 0.44), respectively.

## 4. Discussion

In the present bidirectional Mendelian randomization analysis, we assessed causal associations between CAD and AF. In line with the previous observational studies, our study suggested that CAD mediated at least a portion of the genetic effect on incident AF. However, no evidence demonstrated the causal effect of genetically predicted AF on CAD. 

As early as 1983, the Framingham Study showed that the incidence of CAD patients suffering from AF was higher than that of normal individuals. Clinically, a large proportion of patients have obvious tachyarrhythmia after acute myocardial infarction (AMI). According to the Cardiac Arrhythmias and Risk Stratification After Acute Myocardial Infarction (CARISMA) study, the implantable cardiac monitor in AMI patients documented a 28% incidence of new-onset AF [7]. In an Israeli cohort study including 13,297 acute coronary syndromes, 755 (5.7%) patients developed AF during hospitalization, and the development of AF in this population was evaluated as an independent predictor of mortality for ACS patients [6]. Alexander Romanov et al. reported that 58% AMI patients developed new-onset AF during the follow-up in their prospective observational study, indicating that AF is a frequent arrhythmia after AMI [8]. Our results indicated that genetic variants associated with CAD were significantly associated with incident AF, which is consistent with these observational clinical studies. This seems to give some enlightenment to clinical decision-making. Regular heart-rhythm monitoring such as smart wearable devices, handheld electrocardiograms or implantable loop recorders might be beneficial to combat the expanding incidence of AF in CAD patients.

Previous studies also indicated that the morbidity of CAD increases in AF patients. W S Aronow et al. reported that AF caused a 2.2 times greater incidence of new coronary events [20]. In another study, a sociodemographic-adjusted model demonstrated that AF was associated with about a 2-fold increased risk of myocardial infarction [9]. This increased incidence was more pronounced in women [21]. Both new-onset AF and previous AF are associated with a worse prognosis of ACS [22]. However, contrary to previous research, no evidence suggested that genetic variants associated with AF were related to the increasing incidence of CAD in our present study, indicating that AF is not causative of CAD. One possibility is that the associated comorbidities of AF, such as thrombosis, are the causal link between AF and CAD. Due to the presence of the left atrial appendage, patients with AF are prone to thrombosis. The risk of stroke in patients with AF is approximately five-fold that in healthy individuals [23]. It was reported that platelet activity increased significantly in patients with AF and diabetes [24]. Increasing platelet activity contributes to thrombosis, which might lead to CAD. In another study, the association of D-dimer with CAD was greater in those with AF than in those without AF [25]. Coronary embolism caused by the activation of the coagulation system may also be one of the reasons for the high incidence of ischemic heart disease in patients with AF. Our results suggested that in order to prevent CAD, it might be critical to achieve early anticoagulation in patients with AF. However, more data from randomized clinical trials are still needed to support our hypothesis.

The chief strength of the present investigation is that TSMR analysis was used to exclude confounders and reverse causality. We used three methods, including IVW, MR-Egger, and weighted-median, for TSMR to ensure the robustness of our study. Furthermore, MR-Egger regression analysis was utilized to evaluate the horizontal pleiotropic pathway. However, there are several limitations in our study. First, we used summary-level statistics in our study. Due to the inability to obtain individual clinical data, we cannot perform a specific subtype analysis of CAD. Second, our study mainly included participants of European ancestry, which cannot be generalized to other ethnicities. More data are needed to repeat the analysis in other populations.

## 5. Conclusions

In conclusion, this comprehensive TSMR analysis provides evidence that patients with CAD are associated with an increased risk of AF. However, no causal association was found between patients with AF and the risk of CAD. These findings benefit clinical decision making. Early heart-rhythm monitoring should be performed in patients with CAD. The prevention and treatment of AF complications such as thrombosis may be important to reduce the incidence of CAD in AF patients.

## Figures and Tables

**Table 1:** Intercepts of Mendelian randomization (MR)-Egger regression analysis.

Exposure	Outcome	Intercepts	*p*-Value
CAD	AF	−0.0026	0.675
AF	CAD	0.0054	0.0591

CAD: coronary artery disease; AF: atrial fibrillation.

**Table 2:** Association of genetically predicted CAD with AF.

Method	OR (95% CI)	*p*-Value
IVW	1.11 (1.05, 1.17)	<0.001
MR-Egger	1.14 (1.00, 1.29)	0.049
Weighted-median	1.13 (1.08, 1.19)	<0.001

CAD: coronary artery disease; AF: atrial fibrillation; IVW: inverse-variance weighted.

**Table 3:** Association of genetically predicted AF with CAD.

Method	OR (95% CI)	*p*-Value
IVW	1.01 (0.97, 1.04)	0.76
MR-Egger	0.95 (0.89, 1.02)	0.15
Weighted-median	1.00 (0.95, 1.05)	0.97

AF: atrial fibrillation; CAD: coronary artery disease; IVW: inverse-variance weighted.

## Data Availability

The data presented in this study are available in the public database The MRC IEU OpenGWAS data infrastructure (https://gwas.mrcieu.ac.uk/ accessed on 24 February 2022).

## Supplementary Materials

The following are available online at https://www.mdpi.com/article/10.3390/jcdd9030069/s1, Table S1: Identified instrumental variables for coronary artery disease, Table S2: Identified instrumental variables for atrial fibrillation.
