# Supplementary material for: Coronary Artery Disease and Atrial Fibrillation: A Bidirectional Mendelian Randomization Study
Source: J Cardiovasc Dev Dis. 2022 Feb 27;9(3):69. doi: 10.3390/jcdd9030069 (PMC8949548; doi:10.3390/jcdd9030069)
Supplement: Supplementary file 1 [file jcdd-09-00069-s001.zip › jcdd-1614996-supplementary.pdf]

Table S1 Identified instrumental variables for coronary artery disease.

SNP	Effect allele	Other allele	Eaf	Beta	*p*-value
rs9970807	T	C	0.084903	-0.12575	5.00E-14
rs7528419	G	A	0.21418	-0.11453	1.97E-23
rs6689306	G	A	0.552455	-0.05601	2.60E-09
rs67180937	G	T	0.663052	0.078807	1.01E-12
rs7568458	A	T	0.448518	0.059618	3.62E-10
rs515135	C	T	0.791985	0.067499	3.09E-08
rs17678683	G	T	0.087681	0.098786	3.00E-09
rs115654617	A	C	0.106962	0.137846	3.12E-18
rs16986953	A	G	0.104706	0.08516	1.45E-08
rs1199338	C	A	0.161866	0.073596	3.90E-09
rs4593108	G	C	0.204651	-0.07083	8.82E-10
rs17087335	T	G	0.214637	0.060764	4.59E-08
rs10080815	G	T	0.027558	0.246627	1.33E-15
rs55730499	T	C	0.056243	0.316641	5.39E-39
rs56336142	C	T	0.192738	-0.06681	1.85E-08
rs12202017	G	A	0.300047	-0.06681	1.98E-11
rs9349379	G	A	0.431606	0.131836	1.81E-42
rs11556924	T	C	0.313325	-0.07257	5.34E-11
rs2107595	A	G	0.20047	0.073415	8.05E-11
rs3918226	T	C	0.064515	0.133315	1.69E-09
rs2519093	T	C	0.190872	0.079704	1.19E-11
rs2891168	G	A	0.488668	0.193401	2.29E-98
rs1870634	G	T	0.637485	0.075878	5.55E-15
rs11191416	G	T	0.12747	-0.07925	4.65E-09
rs2487928	A	G	0.418221	0.062633	4.41E-11
rs1412444	T	C	0.369131	0.066812	5.15E-12
rs10840293	A	G	0.549821	0.054714	1.28E-08
rs2128739	C	A	0.676464	-0.06557	7.05E-11
rs2681472	G	A	0.201306	0.074114	6.17E-11
rs11065979	T	C	0.365499	0.068556	1.93E-10
rs11838776	A	G	0.263277	0.068566	1.83E-10
rs10139550	G	C	0.423033	0.05538	1.38E-08
rs8042271	A	G	0.097718	-0.09671	3.68E-08
rs4468572	C	T	0.585831	0.077234	4.44E-16
rs56062135	T	C	0.205729	-0.06974	4.52E-09
rs7212798	C	T	0.146516	0.079961	1.88E-08
rs663129	A	G	0.256835	0.058163	3.20E-08
rs56289821	A	G	0.100378	-0.13361	4.44E-15
rs4420638	G	A	0.166036	0.091906	7.07E-11
rs28451064	A	G	0.121186	0.127571	1.33E-15
rs180803	T	G	0.029268	-0.18092	1.64E-10

Table S2 Identified instrumental variables for atrial fibrillation.

SNP	Effect allele	Other allele	Eaf	Beta	*p*-value
rs11264280	T	C	0.333	0.1347	3.07E-79
rs4073778	A	C	0.5639	0.0486	4.96E-13
rs284277	A	C	0.6174	-0.0422	1.25E-09
rs577676	T	C	0.4383	-0.0923	1.62E-43
rs72694603	T	C	0.3147	-0.0553	2.26E-14
rs79187193	A	G	0.0569	-0.1162	3.15E-14
rs6665642	T	C	0.1176	-0.062	3.06E-08
rs72700114	C	G	0.0756	0.2021	3.29E-54
rs10753933	G	T	0.5518	-0.0609	9.84E-20
rs6689306	G	A	0.5872	-0.046	1.36E-11
rs146518726	A	G	0.0328	0.1605	8.27E-15
rs74832855	G	A	0.0369	0.1216	1.43E-11
rs7529220	C	T	0.8469	0.0621	1.98E-10
rs2885697	T	G	0.6482	-0.0439	2.88E-10
rs6546620	C	T	0.7999	0.0602	3.19E-12
rs28387148	T	C	0.1051	0.0741	6.25E-11
rs6747542	C	T	0.4642	-0.0554	1.10E-16
rs7574892	A	G	0.4847	0.0552	1.98E-16
rs67969609	G	C	0.071	0.0711	1.71E-08
rs35544454	T	A	0.1918	-0.0589	1.10E-11
rs72926475	A	G	0.1228	-0.0683	2.37E-11
rs2288327	G	A	0.1564	0.0919	7.26E-25
rs56326533	C	T	0.3919	0.0685	6.28E-24
rs2540949	T	A	0.3847	-0.0659	2.95E-22
rs4642101	G	T	0.6397	0.0706	2.95E-24
rs6771054	C	T	0.4035	-0.0457	2.42E-11
rs7612445	T	G	0.1879	0.0493	4.81E-09
rs62254082	C	T	0.3865	0.0404	6.34E-09
rs1278493	A	G	0.5645	-0.0389	8.77E-09
rs34080181	A	G	0.379	-0.0446	1.28E-10
rs73041705	C	T	0.2985	-0.0443	1.55E-09
rs60902112	T	C	0.2262	0.0445	1.72E-08
rs6790396	G	C	0.5959	0.0627	2.40E-20
rs10804493	A	G	0.6505	0.0558	1.63E-15
rs6838973	T	C	0.4406	-0.1514	1.03E-111
rs74500426	T	G	0.0764	-0.0921	4.29E-13
rs1906615	T	G	0.1991	0.3658	1.00E-200
rs2739197	G	C	0.2458	0.116	3.20E-41
rs1458038	T	C	0.3087	0.0434	1.74E-09
rs10213171	G	C	0.0609	0.091	1.32E-11

rs10520260	G	A	0.3214	-0.0457	3.36E-10
rs17171711	T	C	0.1775	0.1086	1.95E-35
rs6580277	G	A	0.2369	0.067	1.64E-17
rs337705	G	T	0.3749	0.0564	1.63E-16
rs1838747	G	A	0.4954	0.0391	4.13E-09
rs62377206	A	G	0.0554	0.0846	8.21E-09
rs6882776	A	G	0.2835	-0.0711	9.64E-22
rs6596717	A	C	0.6049	-0.0404	3.00E-09
rs10520002	A	G	0.0988	0.0626	2.85E-08
rs72966339	T	C	0.3679	-0.0616	7.42E-19
rs2031522	G	A	0.3764	-0.0436	1.47E-10
rs34969716	A	G	0.3051	0.0702	1.60E-19
rs3176326	A	G	0.1982	-0.0626	1.42E-13
rs73366713	A	G	0.1396	-0.1035	1.53E-25
rs4946333	G	A	0.4897	0.0639	5.47E-22
rs117984853	T	G	0.1013	0.1228	1.34E-24
rs55734480	A	G	0.2494	0.0548	2.20E-12
rs55985730	G	T	0.06	0.0867	5.24E-09
rs74910854	G	A	0.0693	0.09	4.31E-08
rs56201652	A	G	0.267	-0.0531	1.74E-12
rs7789146	A	G	0.1787	-0.0584	2.12E-11
rs6462079	A	G	0.7208	0.0466	8.79E-10
rs11773845	A	C	0.5856	0.1054	2.39E-55
rs62521286	G	A	0.0663	0.1202	4.50E-19
rs3943207	T	G	0.1167	-0.0638	6.92E-10
rs6994744	C	A	0.4954	0.0405	1.09E-09
rs35963991	T	G	0.1494	0.0525	2.80E-08
rs7508	A	G	0.7109	0.0711	1.69E-21
rs10821415	A	C	0.4132	0.0821	2.92E-34
rs2274115	G	A	0.7003	0.0487	1.69E-10
rs11191116	T	C	0.348	-0.041	4.42E-09
rs7915134	T	C	0.1439	-0.1168	1.42E-34
rs12245149	A	C	0.4739	-0.047	1.66E-12
rs10458662	G	T	0.1722	0.0544	6.93E-10
rs11598047	G	A	0.1621	0.1537	8.95E-66
rs34936990	A	G	0.1207	0.1294	2.95E-37
rs4757877	G	A	0.7552	-0.0723	2.93E-20
rs4935786	A	T	0.7327	-0.0463	4.85E-09
rs76097649	A	G	0.0933	0.1151	1.26E-20
rs10842383	T	C	0.1478	-0.0988	2.88E-25
rs71454237	A	G	0.209	-0.062	1.78E-13
rs12426679	T	C	0.5278	-0.0391	4.95E-09
rs6560886	C	T	0.7884	0.051	1.49E-08
rs883079	T	C	0.7074	0.0981	2.84E-40

rs17380837	T	C	0.307	-0.0501	4.80E-12
rs2860482	C	A	0.726	-0.054	1.21E-12
rs775498	G	A	0.2798	0.0423	1.05E-08
rs10773657	A	C	0.862	-0.0575	2.54E-08
rs35569628	C	T	0.223	-0.0452	1.38E-08
rs9506925	T	C	0.2669	0.0449	2.72E-09
rs74884082	T	C	0.2495	-0.0493	3.48E-10
rs4587869	C	G	0.2849	0.0716	1.19E-20
rs2738413	G	A	0.5049	-0.0778	2.55E-31
rs10141892	C	T	0.5833	-0.0452	2.95E-11
rs28631169	T	C	0.1982	0.0522	5.35E-10
rs7172038	G	T	0.1597	0.112	4.78E-36
rs2759301	A	G	0.4542	0.039	5.04E-09
rs4965430	G	C	0.6136	-0.0441	1.26E-10
rs2359171	A	T	0.176	0.1746	4.65E-91
rs77316573	T	C	0.1991	0.0529	3.26E-09
rs140185678	A	G	0.0351	0.1659	2.43E-14
rs1563304	T	C	0.178	0.0644	2.56E-12
rs4252627	T	C	0.6679	-0.0415	5.63E-09
rs72811294	C	G	0.1131	-0.072	9.67E-12
rs7225165	A	G	0.1133	-0.0655	3.20E-09
rs7224711	T	C	0.5222	-0.0365	3.72E-08
rs9953366	C	T	0.6631	0.049	1.82E-11
rs8088085	C	A	0.4646	-0.0365	4.79E-08
rs2834618	G	T	0.1056	-0.0944	3.41E-17
rs464901	C	T	0.3353	-0.0508	1.53E-12
rs133885	A	G	0.4377	0.0405	2.22E-09
